# Assessment of Motor Imagery and Its Relationship to Functional Impairment in Post-Stroke Patients: A Descriptive Cross-Sectional Study

**DOI:** 10.3390/jcm13195929

**Published:** 2024-10-04

**Authors:** Lidia Martínez-Rolando, Patricia Martín-Casas, Teresa Pérez-Pérez

**Affiliations:** 1 Rey Juan Carlos University Hospital of Móstoles, 28933 Madrid, Spain; martinezrolandolidia@gmail.com; 2 Department of Radiology, Rehabilitation and Physiotherapy, Faculty of Nursing, Physiotherapy and Podiatry, Complutense University of Madrid, Health Research Institute of the San Carlos Clinical Hospital of Madrid (IdISSC), 28040 Madrid, Spain; 3 Department of Statistics and Data Science, Faculty of Statistical Studies, Complutense University of Madrid, 28040 Madrid, Spain; teperez@ucm.es

**Keywords:** assessment, motor imagery, stroke, physical function

## Abstract

**Background:** Motor and cognitive sequelae are common in patients who have experienced a stroke. Recent advances in neuroscience have enabled the development of novel therapeutic approaches, such as motor imagery, which facilitate motor learning. The objective of this study is to examine the relationship between implicit and explicit motor imagery abilities and their correlation with functional impairment in post-stroke patients. **Methods:** A descriptive cross-sectional study was conducted with 36 patients who had experienced a stroke between March 2008 and March 2023. The capacity to generate both implicit and explicit motor imagery and to perform physical functions was evaluated. The relationship between implicit and explicit motor imagery measures was investigated using Pearson’s correlation coefficient. The factorial structure, which encompasses the capacity to generate motor imagery, whether implicit or explicit, and physical function, was subjected to analysis. **Results:** A correlation was identified between the time taken to identify images and the accuracy of this process, with the right hand (R = 0.474), the left hand (R = 0.568), and the left foot (R = 0.344) all demonstrating significant associations. Additionally, a notable correlation was observed between the two subscales of the KVIQ-10 scale (R = 0.749). No association was identified between the capacity to generate implicit and explicit motor imagery. Two- and three-factor solutions were obtained for the right and left hemibodies, respectively. On both sides, accuracy in identifying images and physical function constituted a single factor, while time to generate images for both hands and feet constituted a second factor. **Conclusions:** In conclusion, no significant data were reported regarding the association between the capacity to generate implicit and explicit motor imagery in the studied sample. However, the ability to generate implicit motor imagery was related to physical function, suggesting that it may serve as a screening criterion for implementing specific therapeutic approaches in post-stroke patients.

## 1. Introduction

Stroke is defined as the death of brain cells, spinal cord, or retina and is attributed to an ischemic or hemorrhagic event [1]. Annually, approximately 12 million strokes occur, with 53% of these affecting women and 47% affecting men. Most of these cases occur in individuals under the age of 70, indicating that stroke is no longer a predominant disease in older adults. In 2017, the direct and indirect costs associated with stroke in Europe reached 45 billion euros [2,3,4]. 

The occurrence of motor and cognitive sequelae following this event is a common phenomenon, with a prevalence exceeding 80 million survivors worldwide. A substantial proportion of stroke survivors, between 30% and 66%, fail to achieve a satisfactory recovery of motor function in the upper limbs following rehabilitation [5,6,7]. 

Motor imagery is defined as the mental simulation of movement without the actual physical performance of the movement. This results in activation of the central motor areas that are responsible for the execution of movement. A distinction is made between implicit motor imagery (IMI), which occurs when the subject performs a cognitive task of laterality judgment without voluntarily imagining the movement during mental rotation tasks, and explicit motor imagery (EMI), which occurs when the subject imagines movements or actions without these occurring in the body segment involved, activating the same neural circuits as if such movement were to occur [8,9,10,11]. 

The utilization of motor imagery evocation techniques in post-stroke patients has been demonstrated to facilitate the generation of an internal representation of movement, thereby promoting functional redistribution and modulation of implicit circuits. This approach has been shown to enhance motor learning, performance, and motor skill performance [11,12,13].

Notwithstanding the theoretical advantages of motor imagery programs, the extant studies do not differentiate between IMI and EMI, nor do they implement protocols tailored to the patient’s primary condition [7,12,13]. Some clinical trials and systematic reviews have demonstrated that motor imagery training in conjunction with conventional therapy or a brain-computer interface, when added to conventional rehabilitation, has resulted in notable improvements in motor and balance functions, as well as in gait variables in patients who have experienced a stroke [12,14,15]. In recent years, the development of brain-computer interface training and artificial intelligence has opened up promising avenues for technological evaluation and treatment devices [15]. Nevertheless, to date, there is no definitive evidence to support their use in the functional recovery of upper and lower limbs in post-stroke patients when used in isolation [7,14,16,17].

The primary objective of the present study is to evaluate the relationship between the ability to generate motor imagery (IMI and EMI) and physical function in post-stroke patients. The secondary objectives are to analyze the associations between the sociodemographic and clinical characteristics of the patients, to assess their physical function, and to consider their ability to generate implicit and explicit motor imagery.

The primary hypothesis of the present study is that there is a relationship between the capacity to generate motor imagery (both implicit and explicit) in post-stroke patients and physical function. Consequently, the operative hypotheses are that there is a relationship between the capacity to generate implicit and explicit motor imagery and that there is also a relationship between the capacity to generate motor imagery and physical function in post-stroke patients. 

## 2. Materials and Methods

A cross-sectional descriptive observational study was conducted with 36 patients who had experienced a stroke between March 2008 and March 2023. The study was conducted in accordance with the guidelines for the communication of observational studies set forth by the STROBE Declaration [18], the indications of the Declaration of Helsinki, and the Good Clinical Practice guidelines. The study was approved by the Clinical Research Ethics Committee of the Fundación Jiménez Díaz, and the information obtained was always treated in accordance with current data protection regulations [19,20].

### 2.1. Participants

Subjects were eligible for inclusion in the study if they met the following criteria: history of any type of stroke, age 18 years or older, sensorimotor impairment in upper and/or lower limbs post-stroke, and receipt of rehabilitation treatment within the past two years at a single hospital where the study was conducted. The principal investigator of the study then approached the subjects in person or by telephone to ascertain their willingness to participate. 

Subjects were excluded if they presented concomitant neurological pathology, if they exhibited cognitive impairment (MEC-35 ≤ 23) [12], if they exhibited significant alterations in language comprehension (subtest score of auditory comprehension in the Boston tests below the 35th percentile) [12], if they had a diagnosis of blindness derived from the event or prior to it, or if they had associated comorbidities that limited their evaluation.

### 2.2. Performance Measures

An interview was conducted to collect data on sociodemographic variables (age, sex, educational level, employment status), clinical variables (stroke characteristics, affected hemibody, time elapsed since the event, manual dominance, main risk factors, current medication) and rehabilitation treatment variables (if the patient was undergoing treatment, since when, what type of treatment and its dose). 

**Figure 1:**
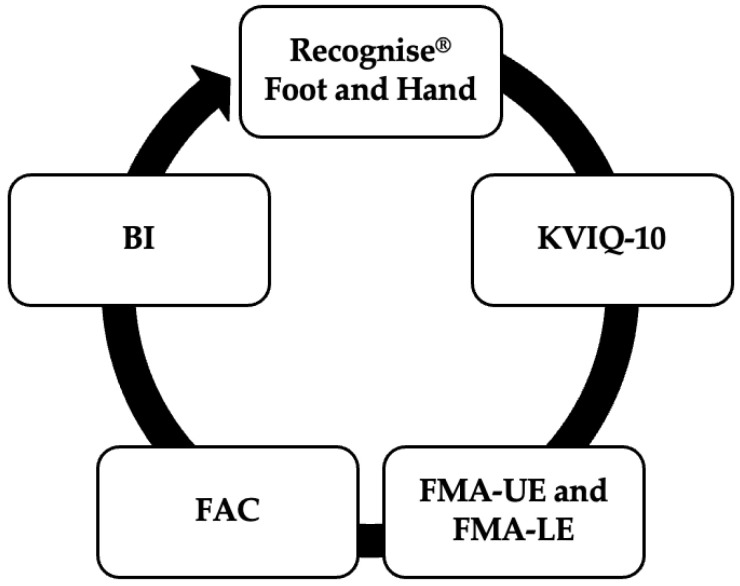
Performance measures. KVIQ-10: Kinesthetic and visual imagination questionnaire; FMA-UE: Fugl–Meyer upper extremity; FMA-LE: Fugl–Meyer lower extremity; FAC: Functional Ambulation Categories; BI: Barthel index.

Performance measures were evaluated using specific and validated tools (Figure 1). 

The capacity to generate IMI was examined using the Recognise^®^ App (version 1.9), developed by the NOI Group^TM^ (Adelaide, Australia). The mean identification time for each image (in seconds) and the accuracy of right or left laterality recognition with rotation (in percentage) were evaluated through 20 images, with a maximum of five seconds allowed per image, for both the upper and lower limbs [10]. 

The capacity to generate explicit motor imagery was assessed using the Spanish version of the Kinesthetic and Visual Imagery Questionnaire (KVIQ-10), which assesses the degree to which patients can visualize or feel an imagined movement through five items in two subscales (visual and kinesthetic) [20,21]. 

A comprehensive evaluation of the physical function of post-stroke patients was conducted using a variety of performance measures. The assessment of upper limb function, lower limb function, walking ability, and level of independence was conducted using the Fugl–Meyer upper extremity (FMA-UE) scale [22,23,24], Fugl–Meyer lower extremity (FMA-LE) scale [23,25], the Functional Ambulation Categories (FAC) scale [26,27,28], and the Barthel index (BI), respectively [29,30]. 

### 2.3. Sample Size and Statistical Analysis

The minimum sample size required to achieve 80% power in a bilateral correlation contrast, assuming an alpha risk of 0.05, a Pearson’s correlation coefficient of 0.5, and a loss rate of 10%, is 33 patients.

A descriptive analysis of the participants’ sociodemographic and clinical characteristics was conducted. Qualitative variables were expressed as frequencies and percentages, accompanied by their 95% confidence intervals. Quantitative variables were summarized as means and standard deviations or median and interquartile ranks, as appropriate. The correlation between IMI and EMI was quantified using Pearson’s correlation coefficient. A high positive coefficient value indicates a significant positive correlation. The factor structure linking the capacity to generate IMI and EMI with functional impairment was examined. A factor analysis was conducted based on a Pearson correlation matrix and using the unweighted least-squares extraction method, which is robust to any deviation from the normal distribution [31]. An eigenvalue of at least 1 was employed to ascertain the number of factors. To substantiate the utilization of factor analysis, a series of tests and measurements were conducted. The correlation matrix indicates the presence of underlying factors that may be shared by the components, as evidenced by the presence of high positive or negative values. Bartlett’s test, which contrasts whether the correlation matrix is significantly different from an identity matrix, is a prerequisite for performing factor analysis if the *p*-value is less than 0.05 [32]. The Kaiser–Meyer–Olkin (KMO) measure is an indicator of the suitability of the data for factor analysis. A value of 0.5 or greater than 0.5 is considered optimal [33]. Finally, the communalities, which represent the proportion of the variance of each variable explained by each factor, were calculated. A higher value of the communalities indicates a more reliable factor analysis. 

The alpha level was set at 0.05 to denote statistical significance. The statistical analysis was performed with IBM SPSS^®^ version 28.0. 

## 3. Results

### 3.1. Sociodemographic and Clinical Characteristics

The study population consisted of 36 patients. The sociodemographic and clinical characteristics of the subjects are presented in Table 1, Table 2 and Table 3. All patients included had experienced a single cerebrovascular accident.

Half of the patients had hypertension, and 75% had dyslipidemia. Additionally, 36% had type II diabetes mellitus and diagnosed heart disease, and 19% had confirmed smoking. Of the total number of patients, 33 (91.7%) exhibited two or more risk factors, while only one patient demonstrated no risk factors.

At the time of the interview, 22 patients (61.1%) were undergoing rehabilitation treatment. Of these, six (27.27%) were receiving interdisciplinary treatment, which was conducted by a team of professionals, including physiotherapists, occupational therapists, speech therapists, and physicians. The mean duration of treatment was 3.11 h (SD = 3.58), with an average length of 3.25 months (SD = 2.83). Among patients undergoing therapy, the longest period was 10 months. The studied sample included patients who were admitted in the acute or subacute phase and were receiving treatment during their hospital stay, as well as those who, once discharged, continued their treatment on an outpatient basis.

### 3.2. Capacity to Generate Both Implicit and Explicit Motor Imagery

In the assessments of IMI, it was observed that the mean recognition time of left-hand images was slightly lower than that of right-hand images (left: 2.19 s, SD = 0.83 vs. right: 2.35 s, SD = 0.73). However, the level of accuracy was higher for the right hand (56.67% for the left hand, with a SD = 25.19, compared to 60.56% for the right hand, with SD = 22.54%). Similarly, the accuracy in recognizing right-foot images with the Recognise Foot^®^ application was higher than that observed in the recognition of left foot images (70.28% for the right foot, with SD = 23.60% vs. 72.22% for the left foot, with SD = 19.58%).

A total of 22 (61.1%) and 26 (72.2%) patients required a response time of ≤2.5 s to identify right and left-hand images, respectively. With regards to accuracy, 13 (36.1%) and 9 (25%) patients demonstrated an accuracy of ≥80% in identifying these images, respectively. About the lower limb, the trend was reversed. Of the participants, 26 (72.2%) and 23 (63.9%) required less than or equal to 2.5 s to identify right- and left-foot images, respectively. In terms of accuracy, 18 (50%) and 19 (52.8%) participants demonstrated an accuracy of 80% or greater, respectively.

No significant differences were identified in terms of image discrimination between one hemibody and the contralateral one, either in terms of time [Diff_hands_ = 0.158; 95%CI (−0.288 to 0.604) or Diff_feet_ = −0.009; 95%CI (−0.356 to 0.339)] or the accuracy (Diff_hands_ = 3.88; 95%CI (−5.342 to 13.120) and Diff_feet_ = 1.94; 95%CI (−4.363 to 8.252)]. However, significant differences were identified regarding the accuracy of anatomical region recognition according to the hemibody [(Diff_right_ = −11.6; 95%CI (−18.060 to −5.272) and Diff_left_ = −13.6; 95%CI (−21.583 to −5.638)]. 

The capacity to generate explicit motor imagery, as evaluated by the KVIQ-10 scale, yielded a mean score of 35.08 points (SD = 12.66). About the two subscales, the patients exhibited, on average, a marginally higher score on the visual scale than on the kinesthetic scale (18.28 points, SD = 6.35 vs. 17.11 points, SD = 7.23). However, this discrepancy is not statistically significant [Diff = −1.167; 95%CI (−2.819 to 0.485)]. 

### 3.3. Correlation between the Variables That Determine the Capacity to Generate Both Implicit and Explicit Motor Imagery, as Well as the Correlation between Both These Abilities

Significant correlations were obtained between the time required to identify images and the accuracy of the right hand [R = 0.474; 95%CI (0.172 to 0.694)] and left hand [R = 0.568; 95%CI (0.294 to 0.755)], as well as in the left foot [R = 0.344; 95%CI (0.018 to 0.605)]. 

The correlation study of the time to distinguish images of hands and feet of both hemispheres yielded significant values between the two anatomical regions of the same hemibody. These values were as follows: R = 0.523; 95%CI right hand—right foot (0.235 to 0.727) and R = 0.655; 95%CI left hand—left foot (0.416 to 0.810). Furthermore, a correlation was identified in the variable time for the differentiation between images of the hands of one hemibody and the contralateral one [R = −0.427; 95%CI right hand—left hand (−0.663 to −0.115)].

Finally, the correlation analysis of the variable “accuracy” to distinguish the right and left images of hands and feet demonstrated significant results, particularly in relation to the accuracy of the two body regions of the same hemibody [R = 0.605; 95%CI right hand—right foot (0.346 to 0.779)] and [R = 0.535; 95%CI left hand—left foot (0.251 to 0.735)]. Additionally, a significant correlation was identified between the accuracy when distinguishing between images of the left and right feet [R = −0.642; 95%CI (0.397 to 0.801)]. 

When the two subscales of the KVIQ-10 scale (visual and kinesthetic) were considered, a significant relationship was observed [R = 0.749; 95%CI (0.557 to 0.865)]. However, no significant data were reported for the association between the capacity to generate implicit and explicit motor imagery (Table 4).

### 3.4. Physical Function of the Sample

The mean value obtained from the evaluation of the upper limb function showed that the sample had a mild mean impairment for the upper limb (92.53 points, SD = 26.07) and a moderate mean impairment for the lower limb (77.97 points, SD = 16.88). 26 patients (72.2%) had mild functional impairment in the upper limb and 21 (58.3%) in the lower limb. Only one patient (2.8%) had severe functional impairment in both the upper and lower limbs. The sample presented a mean score in the sum of both scales of 170.50 points, SD = 40.92. 

Walking ability, quantified with the FAC scale, showed that only one patient did not walk (2.8%), that most patients (30.6%; n = 11) walked independently in the community, that 19.4% (n = 7) walked at home, and that 27.8% (n = 10) were nonfunctional ambulators.

Finally, the sample was found to have an average level of moderate dependence as quantified with the BI (82.22 points, SD = 22.41).

### 3.5. Factor Analysis of Motor Imagery and Physical Function

According to the correlation matrices (Table 5 and Table 6), all variables showed high correlations with at least one of the other variables, except KVIQ-10 on the right hemibody (|r| < 0.20 in all cases).

Examination of the KMO values (0.544 and 0.542 for the right and left hemibodies, respectively) and Barlett’s test of sphericity (*p*-value < 0.001 in both cases) indicated the feasibility of factor analysis.

Factor extractions yielded three factors for both hemibodies with a significant eigenvalue of at least 1.0. However, this solution was not entirely satisfactory for the right side, and a second model was run, eliminating KVIQ-10 due to its low correlation with the other variables. The two-factor solution obtained was easier to interpret. The proportions of the total variance explained were 68.23% (right hemibody) and 74.32% (left hemibody). No variable contributed to more than one factor. 

On both hemibodies, the first factor consisted of 6 variables: hand and foot accuracy, FMA-UE, FMA-LE, FAC, and BI. The second factor consisted of 2 variables: hand and foot time. On the left hemibody, the third factor is essentially just the KVIQ-10. They can be interpreted as Factor 1: “Accuracy and physical function”, Factor 2: “Time”, and Factor 3: “Capacity to generate explicit motor imagery” (Table 7).

## 4. Discussion

The present cross-sectional descriptive study is the first study to analyze the relationship between the capacity to generate implicit and explicit motor imagery and its association with the physical function and sociodemographic and clinical variables in post-stroke patients. We conducted a comprehensive assessment of physical function in post-stroke patients using a variety of performance measures to assess upper and lower limb function, walking ability, and level of independence. 

The results suggest that there is an association between the variables that determine the capacity to generate IMI, both in body regions and between hemibodies. In addition, accuracy in identifying motor imagery is associated with physical function in post-stroke patients. However, no relationship is found between the capacity to generate EMI and physical function, nor between the capacity to generate IMI and EMI.

Thirty-six patients were evaluated with a mean age of 66.78 years (SD 9.87 years) were evaluated. This sample size is larger than that of similar studies, such as that of Vries et al., [11] who evaluated 16 patients with a mean age of 51.38 years (SD 10.03) [11]. In this sense, the mean age of the patients indicates, as stated by the World Health Organization, that stroke is no longer a predominant event in the elderly [2]. 

It should be noted that no previous studies have been conducted using the Recognise^®^ tool in post-stroke patients. However, research has been found in samples with chronic pain or dystonic component, where it was concluded that there was a decrease in response time if the patient took more than 2.5 s to respond, that there was a difference in response time between one side and the contralateral side if there was a difference of more than 0.3 s, and that there was a decrease in the accuracy of left/right judgments if it was less than 80% [10,34,35]. 

Based on these cut-off points, it can be said that the results of this study indicate that patients evaluated after stroke did not show a mean increase in response time to discriminate images of both hands and feet, nor were there significant differences on one side compared to the contralateral side. However, they did show a reduction in the accuracy of laterality judgments of both hands and feet. However, these differences were not significant when comparing the results of one hemibody and the contralateral one. In this sense, authors such as Díaz López et al. and Razmus and Raimo et al. suggest that after having experienced a stroke, there could be an affectation of the body schema, manifested by a deficit in the imagination of one’s own movements and the execution of mental rotation due to motor impairment and suppression of reflex activity [12,36,37].

The results of this study, using the KVIQ-10 scale, suggest that patients have a better capacity to generate explicit visual motor imagery than kinesthetic imagery, but this difference is not significant (*p* = 0.161). Other studies, such as the one conducted by Melogno et al. using the 20-item scale, found that Spanish-speaking patients were more adept at visual imagery compared to the kinesthetic modality [22].

It should be noted that authors such as Emerson et al. suggest that cognitive impairment resulting from stroke may condition the ability to follow verbal instructions, which may translate into difficulty generating motor imagery in a clinical setting. Therefore, this consideration should be considered when interpreting the results of the assessment of the capacity to generate EMI [38].

The mean total score of both scales (FMA-UE and FMA-LE) in this study was 170.50 points (SD 40.92); this result is similar to those of the study by Nilsson et al. with the same sample size (n = 36) before the start of an intervention protocol, who found a mean score of 175.5 points (SD 36.9) [39]. Despite finding similar scores, differences were found in walking ability, with a median of 3.5 in the present study (most patients were able to walk independently in the community) and a median of 0 in the Nilsson et al. study (most patients were unable to walk) [39]. This similarity in upper and lower limb physical function and the difference in walking ability may be due to the fact that Nilsson et al. included patients who were in the acute and subacute phase (first 8 weeks after the event), and the present study also included patients who were in the chronic phase who had already received and/or were receiving treatment to improve functional performance in activities of daily living such as walking [40].

This study found average levels of moderate dependency that are consistent with those found by Díaz López et al. in their clinical study [12]. It is relevant to consider that researchers such as Onabajo et al. have suggested that the achievement of functional independence after suffering a stroke is largely determined by good balance function, independent of sociodemographic factors such as age or gender [41]. In this sense, both this study and the study by Díaz López et al. were performed in a subgroup of patients whose baseline characteristics predisposed them to a better functional status, which would explain the scores obtained [12].

This analysis reported that there was no relationship between the capacity to generate IMI and EMI, a conclusion also reached by Vries et al. in stroke patients and Jeannerod et al. in a healthy population [11,42]. These findings highlight the importance of detailed assessment. The absence of a significant change in the capacity to generate IMI could favor the implementation of treatment programs that include tasks of laterality judgment and mental rotation generation, as noted by Díaz López et al. [12].

Data analysis reported the association between accuracy in performing cognitive tasks of judging laterality and patients’ physical function. Amesz et al. investigated this relationship and also found an association (R2 = 0.21; *p* = 0.009) for the upper limb [43,44]. Knowing the factors that determine the physical function of patients who have experienced a stroke is a priority, since only 12% achieve a complete functional recovery of the upper limb. The specific characteristics of the sample studied, including patients with different types of stroke affecting different brain structures and a higher-than-usual prevalence of cerebellar and basal ganglia strokes, may limit the generalizability of the results. The inclusion and exclusion criteria and the specific characteristics of the clinical setting in which the study was conducted may have influenced these results. These factors should be considered when interpreting and applying our findings.

The convenience sampling method resulted in a homogeneous subgroup in terms of capacity to generate motor imagery with relatively good physical function despite the lack of a time limit since the event occurred. It should also be noted that this is the first study to include a detailed analysis of both the upper and lower limbs, and that there is a paucity of literature using tools such as the Recognise^®^ App (version 1.9) for this population. 

As a descriptive cross-sectional study, it cannot be assumed that the associations found in all analyses are causal. Further studies with larger samples are needed to better understand the potential role of motor imagery in post-stroke rehabilitation.

Our findings highlight the need for a thorough assessment of the capacity to generate motor imagery in individuals who have experienced a stroke, using specific tools to assess IMI and EMI. This approach could lead to a more precise selection of tools for integrating motor imagery training with conventional treatments, as emerging evidence suggests that this combination may be more effective [12,14,15,16,17]. Rapid advancements in digital devices and artificial intelligence are currently transforming rehabilitation models by enabling more intensive and effective treatments for post-stroke patients [44]. A deeper understanding of the role of motor imagery in motor learning in diverse populations may lead to improved outcomes, and further research with larger and more diverse samples is urgently needed.

## 5. Conclusions

A relationship between implicit and explicit motor imagery has not been found, so both assessments should be performed to analyze the capacity of the post-stroke patient to generate motor imagery. Furthermore, the capacity to generate IMI is related to physical function and may be a screening criterion for the implementation of specific therapeutic approaches in post-stroke patients. 

## Figures and Tables

**Table 1:** Sociodemographic characteristics of the sample (n = 36).

	Age (Years)	Time Since Event (Months)		Sex		Education Level				Employment Situation				
				Man	Woman	Uneducated	Basic Education	High School	University School	Active	Unemployed with Benefits	Unemployed without Benefits	Pensioner	Retired
Mean (SD)	66.78 (9.87)	19.22 (31.33)	Frecuency (n)	25	11	1	19	13	3	4	6	1	8	17
Minimum–Maximum	45–82	0–180	Percentage	69.4	30.6	2.8	52.8	36.1	8.3	11.1	16.7	2.8	22.2	47.2

SD: Standard Deviation.

**Table 2:** Clinic characteristics of the sample (n = 36).

	Stroke Type		Anatomical Location									
	Ischemic	Hemorrhagic	Protuberance	Cerebellum	Parietal Region	Posterior Cerebral Artery	Middle Cerebral Artery	Basal Ganglia	Internal Capsule	Frontoparietal Region	Medulla Oblongata	Thalamus
Frecuency (n)	25	11	1	19	13	3	2	7	6	2	1	2
Percentage	69.4	30.6	2.8	52.8	36.1	8.3	19.4	16.7	5.6	5.6	2.8	2

**Table 3:** Clinic characteristics of the sample in relation to the laterality (n = 36).

	Affected Hemibody		Manual Dominance		
	Right	Left	Right	Left	Ambidextrous
Frecuency (n)	13	23	33	1	2
Percentage	36.1	63.9	91.7	2.8	5.6

**Table 4:** Pearson’s correlation coefficient between the capacity to generate implicit and explicit motor imagery (n = 36).

		Explicit Motor Imagery					
		Visual		Kinesthetic	Visual + Kinesthetic		
		r	*p*-Value	r	*p*-Value	r	*p*-Value
Implicit motor imagery	Right-hand time	−0.058	0.738	−0.115	0.506	−0.087	0.614
	Right-hand accuracy	−0.003	0.986	−0.049	0.775	−0.029	0.865
	Left-hand time	0.026	0.882	−0.031	0.859	−0.011	0.951
	Left-hand accuracy	−0.123	0.476	−0.192	0.261	−0.166	0.334
	Right-foot time	−0.073	0.673	−0.015	0.931	−0.031	0.859
	Right-foot accuracy	−0.194	0.258	−0.139	0.419	−0.186	0.278
	Left-foot time	−0.187	0.274	−0.200	0.241	−0.223	0.191
	Left-foot accuracy	−0.239	0.161	−0.198	0.248	−0.252	0.138

**Table 5:** Correlation matrix of implicit motor imagery of the right hemibody, explicit motor imagery, and physical function. * *p* < 0.05.

		Right-Hand Time	Right-Hand Accuracy	Right-Foot Time	Right-Foot Accuracy	KVIQ-10	FMA-UE	FMA-LE	FAC	BI
IMI (right hemibody)	Right-hand time	1.00	0.474 *	0.523 *	0.086	−0.085	−0.005	−0.051	−0.061	−0.031
	Right-hand accuracy		1.000	0.176	0.605 *	−0.031	0.441 *	0.386 *	0.271	0.433 *
	Right-foot time			1.000	−0.052	−0.030	0.144	0.090	−0.156	0.076
	Right-foot accuracy				1.000	−0.189	0.627 *	0.480 *	0.361 *	0.386 *
EMI	KVIQ-10					1.000	0.082	0.081	−0.133	−0.012
Physical function	FMA-UE						1.000	0.807 *	0.517 *	0.562 *
	FMA-LE							1.000	0.643 *	0.628 *
	FAC								1.000	0.842 *
	BI									1.000

IMI: implicit motor imagery; EMI: explicit motor imagery; KVIQ-10: Kinesthetic and visual imagination questionnaire; FMA-UE: Fugl–Meyer upper extremity; FMA-LE: Fugl–Meyer lower extremity; FAC: Functional Ambulation Categories; BI: Barthel index.

**Table 6:** Correlation matrix of implicit motor imagery of the left hemibody, explicit motor imagery, and physical function. * *p* < 0.05.

		Left-Hand Time	Left-Hand Accuracy	Left-Foot Time	Left Time Accuracy	KVIQ-10	FMA-UE	FMA-LE	FAC	BI
IMI (left hemibody)	Left-hand time	1.00	0.568 *	0.655 *	0.228	−0.011	0.062	−0.028	0.086	−0.036
	Left-hand accuracy		1.000	0.176	0.535 *	−0.169	0.351 *	0.314 *	0.367 *	0.363 *
	Left-foot time			1.000	0.344 *	−0.221	−0.033	−0.140	−0.072	−0.167
	Left-foot accuracy				1.000	−0.253	0.301 *	0.214	0.261	0.237
EMI	KVIQ-10					1.000	0.082	0.081	−0.133	−0.012
Physical function	FMA-UE						1.000	0.807 *	0.517 *	0.562 *
	FMA-LE							1.000	0.643 *	0.628 *
	FAC								1.000	0.842 *
	BI									1.000

IMI: implicit motor imagery; EMI: explicit motor imagery; KVIQ-10: Kinesthetic and visual imagination questionnaire; FMA-UE: Fugl–Meyer upper extremity; FMA-LE: Fugl–Meyer lower extremity; FAC: Functional Ambulation Categories; BI: Barthel index.

**Table 7:** Factor matrix of the right hemibody and left hemibody. * Items with the highest weight and intercorrelation.

Right Hemibody Factorial Matrix			Left Hemibody Factorial Matrix			
Factor			Factor			
	1. Accuracy and Physical Function	2. Time		1. Accuracy and Physical Function	2. Time	3. Capacity to Generate Explicit Motor Imagery
Hand time	0.111	0.923 *	Hand time	0.273	0.896 *	0.372
Hand accuracy	0.606 *	0.432	Hand accuracy	0.586 *	0.421	−0.081
Foot time	0.088	0.495 *	Foot time	0.048	0.687 *	−0.043
Foot accuracy	0.637 *	0.076	Foot accuracy	0.451 *	0.360	−0.329
KVIQ-10	-	-	KVIQ-10	−0.076	−0.217	0.496 *
FMA-UE	0.821 *	−0.035	FMA-UE	0.746 *	−0.162	0.175
FMA-LE	0.831 *	−0.134	FMA-LE	0.813 *	−0.279	0.225
FAC	0.738 *	−0.273	FAC	0.797 *	−0.167	−0.130
BI	0.789 *	−0.143	BI	0.795 *	−0.280	−0.094

KVIQ-10: Kinesthetic and visual imagination questionnaire; FMA-UE: Fugl–Meyer upper extremity; FMA-LE: Fugl–Meyer lower extremity; FAC: functional ambulation categories; BI: Barthel index.

## Data Availability

The original contributions presented in the study are included in the article, further inquiries can be directed to the corresponding author.

## References

[1] Sacco R.L., Kasner S.E., Broderick J.P., Caplan L.R., Connors J.J., Culebras A., Elkind M.S., George M.G., Hamdan A.D., Higashida R.T. (2013). An Updated Definition of Stroke for the 21st Century: A Statement for Healthcare Professionals From the American Heart Association/American Stroke Association. Stroke.

[2] (2022). World Stroke Organization (WSO): Global Stroke Fact Sheet. https://www.world-stroke.org/news-and-blog/news/wso-global-stroke-fact-sheet-2022.

[3] Hernández P.S., Guiu-Guia J.M., Meléndez T.H., Azcárraga P.A. (2022). Logros y retos en la atención del ictus en españa: Desde la estrategia del sistema nacional de salud al plan de acción europeo 2018–2030. Rev. Española Salud Pública.

[4] Wafa H.A., Wolfe C.D.A., Emmett E., Roth G.A., Johnson C.O., Wang Y. (2020). Burden of Stroke in Europe: Thirty-Year Projections of Incidence, Prevalence, Deaths, and Disability-Adjusted Life Years. Stroke.

[5] Einstad M.S., Saltvedt I., Lydersen S., Ursin M.H., Munthe-Kaas R., Ihle-Hansen H., Knapskog A.-B., Askim T., Beyer M.K., Næss H. (2021). Associations between post-stroke motor and cognitive function: A cross-sectional study. BMC Geriatr..

[6] Duncan P.W., Bushnell C., Sissine M., Coleman S., Lutz B.J., Johnson A.M., Radman M., Pvru Bettger J., Zorowitz R.D., Stein J. (2021). Comprehensive Stroke Care and Outcomes: Time for a Paradigm Shift. Stroke.

[7] Villa-Berges E., Laborda Soriano A.A., Lucha-López O., Tricas-Moreno J.M., Hernández-Secorún M., Gómez-Martínez M., Hidalgo-García C. (2023). Motor Imagery and Mental Practice in the Subacute and Chronic Phases in Upper Limb Rehabilitation after Stroke: A Systematic Review. Occup. Ther. Int..

[8] Benito Villalvilla D., López de Uralde Villanueva I., Ríos León M., Álvarez Melcón Á.C., Martín Casas P. (2021). Eficacia de la imagen motora en la esclerosis múltiple: Revisión sistemática. Rev. Neurol..

[9] Moseley G.L. (2012). The Graded Motor Imagery Handbook.

[10] Osuagwu B.A., Vuckovic A. (2014). Similarities between explicit and implicit motor imagery in mental rotation of hands: An EEG study. Neuropsychologia.

[11] De Vries S., Tepper M., Feenstra W., Oosterveld H., Boonstra A.M., Otten B. (2013). Motor imagery ability in stroke patients: The relationship between implicit and explicit motor imagery measures. Front. Hum. Neurosci..

[12] Díaz-López N., Monge-Pereira E., Jodra-Centeno E., Molina-Rueda F., Miangolarra-Page J.C. (2022). Use of recognition of laterality through implicit motor imagery for the improvement of postural control and balance in subacute stroke patients: A randomized controlled study. Rev. Neurol..

[13] Ietswaart M., Johnston M., Dijkerman H.C., Joice S., Scott C.L., MacWalter R.S., Hamilton S.J. (2011). Mental practice with motor imagery in stroke recovery: Randomized controlled trial of efficacy. Brain.

[14] Zhao L.J., Jiang L.H., Zhang H., Li Y., Sun P., Liu Y., Qi R. (2023). Effects of Motor Imagery Training for Lower Limb Dysfunction in Patients With Stroke: A Systematic Review and Meta-analysis of Randomized Controlled Trials. Am. J. Phys. Med. Rehabil..

[15] Kruse A., Suica Z., Taeymans J., Schuster-Amft C. (2020). Effect of brain-computer interface training based on non-invasive electroencephalography using motor imagery on functional recovery after stroke—A systematic review and meta-analysis. BMC Neurol..

[16] Aprigio D., Bittencourt J., Ramim M., Marinho V., Brauns I., Fernandes I., Ribeiro P., Velasques B., E Silva A.C.A. (2022). Can mental practice adjunct in the recovery of motor function in the upper limbs after stroke? A systematic review and meta-analysis. Brain Circ..

[17] Fernandez-Gomez E., Sanchez-Cabeza A. (2018). Motor imagery: A systematic review of its effectiveness in the rehabilitation of the upper limb following a stroke. Rev. Neurol..

[18] Von Elm E., Altman D.G., Egger M., Pocock S.J., Gøtzsche P.C., Vandenbroucke J.P. (2008). The Strengthening the Reporting of Observational Studies in Epidemiology (STROBE) statement: Guidelines for reporting observational studies. J. Clin. Epidemiol..

[19] (2013). World Medical Association Declaration of Helsinki: Ethical Principles for Medical Research Involving Human Subjects. JAMA.

[20] Reglamento (UE) 2016/679 del Parlamento Europeo y del Consejo—De 27 de abril de 2016—Relativo a la Protección de las Personas Físicas en lo que Respecta al Tratamiento de Datos Personales y a la Libre Circulación de estos Datos y por el que se Deroga la Directiva 95/46/CE (Reglamento General de Protección de Datos). https://eur-lex.europa.eu/legal-content/ES/TXT/?uri=celex%3A32016R0679.

[21] Moreno-Verdú M., Martín-Casas P. (2023). Test-Retest Reliability and Criterion Validity of the Spanish Version of Two Motor Imagery Questionnaires in People with Parkinson Disease. J. Neurol. Phys. Ther..

[22] Melogno M., Nunez S., Ubillos S. (2014). Cuestionario de Imaginación Cinestésica y Visual (KVIQ) Procedimiento de Aplicación. Master’s Thesis.

[23] Melogno M., Nunez S., Ubillos S. (2022). Kinesthetic and Visual Motor Imagery in Spanish Healthy and Stroke People. PsyArXiv.

[24] Sullivan K.J., Tilson J.K., Cen S.Y., Rose D.K., Hershberg J., Correa A., Gallichio J., McLeod M., Moore C., Wu S.S. (2011). Fugl-Meyer Assessment of Sensorimotor Function After Stroke: Standardized Training Procedure for Clinical Practice and Clinical Trials. Stroke.

[25] Platz T. (2005). Reliability and validity of arm function assessment with standardized guidelines for the FugI-Meyer Test, Action Research Arm Test and Box and Block Test: A multicentre study. Clin. Rehabil..

[26] Park J., Lee N., Cho M., Kim D., Yang Y. (2015). Effects of mental practice on stroke patients’ upper extremity function and daily activity performance. J. Phys. Ther. Sci..

[27] Hernández E.D., Forero S.M., Galeano C.P., Barbosa N.E., Sunnerhagen K.S., Alt Murphy M. (2021). Intra- and inter-rater reliability of Fugl-Meyer Assessment of Lower Extremity early after stroke. Braz. J. Phys. Ther..

[28] Mehrholz J., Wagner K., Rutte K., Meiβner D., Pohl M. (2007). Predictive Validity and Responsiveness of the Functional Ambulation Category in Hemiparetic Patients After Stroke. Arch. Phys. Med. Rehabil..

[29] Shah S., Vanclay F., Cooper B. (1989). Improving the sensitivity of the Barthel Index for stroke rehabilitation. J. Clin. Epidemiol..

[30] Quinn T.J., Langhorne P., Stott D.J. (2011). Barthel Index for Stroke Trials: Development, Properties, and Application. Stroke.

[31] Douglas L.J., Green P.E. (2003). Analizing Multivariate Data.

[32] Bartlett M.S. (1951). The Effect of Standardization on a χ2 Approximation in Factor Analysis. Biometrika.

[33] Kaiser H.F. (1974). An index of factorial simplicity. Psychometrika.

[34] Bray H., Moseley G.L. (2011). Disrupted working body schema of the trunk in people with back pain. Br. J. Sports Med..

[35] Fiorio M., Tinazzi M., Aglioti S.M. (2006). Selective impairment of hand mental rotation in patients with focal hand dystonia. Brain.

[36] Razmus M. (2017). Body representation in patients after vascular brain injuries. Cogn. Process.

[37] Raimo S., Boccia M., Di Vita A., Iona T., Cropano M., Ammendolia A., Colao R., Angelillo V., Maiorino A., Guariglia C. (2022). Body Representation Alterations in Patients with Unilateral Brain Damage. J. Int. Neuropsychol. Soc..

[38] Emerson J.R., Binks J.A., Scott M.W., Kenny R.P., Eaves D.L. (2018). Combined action observation and motor imagery therapy: A novel method for post-stroke motor rehabilitation. AIMS Neurosci..

[39] Nilsson L., Carlsson J., Danielsson A., Fugl-Meyer A., Hellström K., Kristensen L., Sjölund B., Sunnerhagen K.S., Grimby G. (2001). Walking training of patients with hemiparesis at an early stage after stroke: A comparison of walking training on a treadmill with body weight support and walking training on the ground. Clin. Rehabil..

[40] Vincent-Onabajo G., Joseph E., Musa H.Y. (2018). Impact of balance on functional independence after stroke: A cross-sectional study at rehabilitation settings in Nigeria. NeuroRehabilitation.

[41] Jeannerod M. (2001). Neural Simulation of Action: A Unifying Mechanism for Motor Cognition. NeuroImage.

[42] McAvinue L.P., Robertson I.H. (2007). Relationship between Visual and Motor Imagery. Percept. Mot. Skills.

[43] Amesz S., Tessari A., Ottoboni G., Marsden J. (2016). An observational study of implicit motor imagery using laterality recognition of the hand after stroke. Brain Inj..

[44] Tang E., Moran N., Cadman M., Hill S., Sloan C., Warburton E., Guideline Committee (2024). Stroke rehabilitation in adults: Summary of updated NICE guidance. BMJ.

